# Successful vaccines for naturally occurring protozoal diseases of animals should guide human vaccine research. A review of protozoal vaccines and their designs

**DOI:** 10.1017/S0031182013002060

**Published:** 2014-01-28

**Authors:** MILTON M. MCALLISTER

**Affiliations:** University of Adelaide, School of Animal & Veterinary Sciences, Roseworthy, SA 5371, Australia

**Keywords:** Protozoal diseases, attenuation, vaccination, efficacy, review, animal models of human disease, malaria, one medicine, apicomplexa

## Abstract

Effective vaccines are available for many protozoal diseases of animals, including vaccines for zoonotic pathogens and for several species of vector-transmitted apicomplexan haemoparasites. In comparison with human diseases, vaccine development for animals has practical advantages such as the ability to perform experiments in the natural host, the option to manufacture some vaccines *in vivo*, and lower safety requirements. Although it is proper for human vaccines to be held to higher standards, the enduring lack of vaccines for human protozoal diseases is difficult to reconcile with the comparatively immense amount of research funding. Common tactical problems of human protozoal vaccine research include reliance upon adapted rather than natural animal disease models, and an overwhelming emphasis on novel approaches that are usually attempted in replacement of rather than for improvement upon the types of designs used in effective veterinary vaccines. Currently, all effective protozoal vaccines for animals are predicated upon the ability to grow protozoal organisms. Because human protozoal vaccines need to be as effective as animal vaccines, researchers should benefit from a comparison of existing veterinary products and leading experimental vaccine designs. With this in mind, protozoal vaccines are here reviewed.

## INTRODUCTION

Effective vaccines are available for many protozoal diseases of animals (see Tables 1–4). In contrast, no vaccine is widely available for any protozoal disease of humans, despite great need and considerable effort. Some of the more important protozoal diseases of humans for which vaccines could be invaluable include falciparum malaria, vivax malaria, Chagas' disease, African trypanosomiasis, visceral leishmaniasis, cutaneous leishmaniasis, cryptosporidiosis, and giardiasis.

**Table 1.** Protozoal immunizations with live virulent organisms using controlled-exposure or infection-and-treatment protocols

Disease target	Pathogen	Content	Inoculation	Immunizing procedure	Producers	Production	Notes	Selected citations
Human cutaneous leishmaniasis	*Leishmania major*	Promastigotes	1 × ID	Infect a cosmetically unimportant skin site (leishmanization)	Government sponsored labs in Central Asia	*In vivo* in mice, followed by broth culture	Recent use in Uzbekistan. Skin lesion may endure months	(Dunning, 2009)
Coccidiosis of chickens and turkeys	Numerous species of *Eimeria*	Oocysts	1 × PO via feed, water, spraying chicks, or in ovo	Controlled dose exposure, in the past often followed by a low level of coccidiostat in feed or water	Numerous bio-pharmaceutical corporations	*In vivo* in chickens and turkeys	Widespread use since 1952. Experiments and field trials show improved growth in vaccinated birds	(Long *et al.* 1982; Williams, 2002)
East Coast fever of cattle	*Theileria parva*	Sporozoites of one or more strains	1 × SC	Simultaneous application of vaccine and depot oxytetracycline	VetAgro Tanzania Ltd., & government sponsored labs	Homogenates from infected ticks	Protection endures ≥ 3 years	(Radley *et al.* 1975; Uilenberg, 1999; Di Giulio *et al.* 2009)

**Table 2.** Protozoal vaccines that contain live attenuated organisms

Disease target	Pathogen	Content	Inoculation	Producers	Production	Availability	Notes	Selected citations
Toxoplasmosis abortion in sheep	*Toxoplasma gondii*	Tachyzoites of the S48 strain	1 × SC in pre-breeding ewe lambs	MSD Animal Health, Toxovax	*In vitro* culture in mammalian cells	New Zealand and parts of Europe	Enduring protection. Initial attenuation by rapid passage in mice. Can't encyst. In use since 1988	(Wilkins *et al.* 1988; Buxton *et al.* 1993)
Bovine tropical theileriosis	*Theileria annulata*	Macro-schizonts	1 × SC	Primarily government sponsored labs	*In vitro* culture in lymphoid or monocytic cells	e.g. Turkey, China, India, Iran, Israel, Spain	Enduring protection under field conditions. Attenuation achieved several times by adapting to cell culture. Vaccine organisms cannot be transmitted to ticks	(Hashemi-Fesharki, 1988; Pipano, 1997; Hall *et al.* 1999; Yin *et al.* 2008)
Bovine babesiosis	*Babesia bovis, B. bigemina*	Merozoites	1 × SC or IM in juvenile calves	Primarily government sponsored labs	*In vivo* in splenectomized calves or *in vitro* in bovine erythrocytes	e.g. Australia, Argentina, Columbia, Cuba, Israel	An epidemiologic success, but several vaccines can induce disease (2% vaccine reactions in Cuba) and are contraindicated in adults. An *in vitro* produced vaccine used in Argentina appears to be well attenuated	(Callow *et al.* 1997; Bock and de Vos, 2001; Gerdts and Ortíz, 2004; Echaide, 2008)
Poultry coccidiosis	Numerous species of *Eimeria*	Oocysts	1 × PO via feed, water, spraying chicks	Numerous bio-pharmaceutical corporations	*In vivo* in chickens and turkeys	Used worldwide	‘Precocious’ strains select short prepatency, produce fewer oocysts with reduced virulence but greater production cost	(Jeffers, 1975; McDonald and Shirley, 2009)

**Table 3.** Protozoal vaccines that contain whole killed organisms

Disease target	Pathogen	Content	Inoculation	Producers	Production	Notes	Selected citations
Bovine trichomoniasis	*Tritrichomonas foetus*	Killed trophozoites in adjuvant	2 × SC annually before breeding season	Boehringer – Ingelheim, Trichguard	Axenic culture in broth	Epidemiological meta-analysis and challenge trials support modest to moderate efficacy in females. Short duration of immunity may be sufficient for seasonal breeding. Value in males is unclear	(Cobo *et al.* 2004; Baltzell *et al.* 2013)
Canine giardiasis	*Giardia lamblia*	Killed disrupted trophozoites in adjuvant	2 × SC, then annually	Zoetis, GiardiaVax	Axenic culture in broth	In an experimental challenge within weeks of vaccination, significant reduction of infection and diarrhoea occurred in vaccinates. A trial in an animal shelter did not show a benefit	(Olson *et al.* 1997; Lehmann and Lehmann, 2004)
Equine protozoal myelitis	*Sarcocystis neurona*	Killed organisms in adjuvant	2 × IM	Out of production	*In vitro* culture in mammalian cells	Did not progress beyond conditional licence. There was no good disease challenge model and no published epidemiological analysis to support efficacy	(Witonsky *et al.* 2004)
Neosporosis abortion in cattle	*Neospora caninum*	Killed tachyzoites in adjuvant	2 × SC, then annually	Out of production	*In vitro* culture in mammalian cells	Low overall efficacy. Goals may have been confounded by confusion between prevention of infection in naive cows *vs* prevention of recrudescence in latently infected cows	(Weston *et al.* 2012)

**Table 4.** Protozoal vaccines that contain defined antigens or antigen extracts

Disease target	Pathogen	Content	Inoculation	Manufacturer	Production	Notes	Selected citations
Canine visceral leishmaniasis (in Brazil)	*Leishmania infantum* (*L. chagasi*)	Fucose mannose ligand glycoprotein complex, with saponin adjuvant	3 × SC, then annually	Zoetis, Leishmune	Ag extracted from *in vitro* axenic culture of *L. donovani* (not *L. infantum*)	Field trials support efficacy in reducing prevalence of infection and disease in dogs. Injection reactions common but not serious. Epidemiological evidence of associated reduction of vector-borne zoonotic transmission in Brazil	(Borja-Cabrera *et al.* 2002; Nogueira *et al.* 2005; Palatnik-de-Sousa *et al.* 2009)
Canine visceral leishmaniasis (in Europe)	*Leishmania infantum*	Excreted secreted proteins with saponin adjuvant	3 × SC, then annually	Virbac, CaniLeish	Ag extracted from *in vitro* axenic culture of *L.infantum*	Pre-licensed formulations (with muramyl dipeptide adjuvant) protected dogs from infectious challenge 8 m after vaccination, and in a field trial showed 92% efficacy over 2 years	(Lemesre *et al.* 2005; Lemesre *et al.* 2007)
Canine visceral leishmaniasis (in Brazil)	*Leishmania infantum* (*L. chagasi*)	A2 amastigote ag linked to a histidine tag, with saponin adjuvant	3 × SC, then annually	Hertape Calier, Leish-Tec	Recombinant ag produced by transfected *E. coli*.	Licensed in Brazil since 2007. Partial protection in 7 dogs demonstrated in severe IV challenge administered 4 weeks after vaccination. No further information regarding lab or field studies	(Fernandes *et al.* 2008)
Coccidiosis of chickens	*Eimeria* spp.	*E. maxima* gam56 and gam82 ag with oil-in-water adjuvant	2 × IM in breeding hens before laying	Philbro Animal Health Corp., Coxabic	*In vivo* growth of *E. maxima* in chickens. Gametocyte ag affinity purified from infected gut	Gametocyte ag induces interspecific protection. Maternal immunity is boosted by natural exposure of hatchlings to coccidia. Each vaccine dose is relatively costly, but passive immunity extends to many eggs/chicks. Efficacy supported by challenge experiments and production data	(Sharman *et al.* 2010)
Canine babesiosis	*Babesia canis, B. rossi*	Soluble parasite ag in saponin adjuvant	2 × SC, then twice annually	Out of production	*In vitro* in canine RBCs. Ag extracted from culture supernatant	Mild-moderate reduction of disease severity in vigorous IV heterologous challenge. Injection site reactions reported. No published epidemiological investigations	(Schetters *et al.* 2001; Schetters *et al.* 2006; Freyburger *et al.* 2011)

In comparison to animal vaccine research, human vaccine research has an extra high hurdle in needing to test vaccine efficacy within humans themselves. Nevertheless, this problem is not insurmountable, and numerous highly credible experiments and field trials have been performed on humans (Armijos *et al.* 2003; Roestenberg *et al.* 2009; Olotu *et al.* 2013; Seder *et al.* 2013). So why is it that protozoal vaccines are only available for animals? A number of factors are suggested later in this review.

Indeed, several effective veterinary vaccines do have designs that should be suitable for human vaccines. Vaccine designs that have had repetitive veterinary successes should be considered for adaptation for human vaccines. Protozoal vaccine scientists should familiarize themselves with the comparative performance records of the various design categories of experimental and commercial vaccines, and in particular they should analyse the properties and production methods of the best vaccines that have been used to combat animal diseases.

The following sections of this analytical review article describe the main classes of veterinary protozoal vaccine designs. Illustrative details are provided of the composition and performance of several vaccines, most of which are in current use; readers who prefer less detail may skip to the short descriptions listed in Tables 1–4. Experimental protozoal vaccines are then presented in two sections, the first describing experimental vaccines with good evidence of efficacy, and the second analysing several prominent types of experimental malaria vaccines; these are summarized in Tables 5 and 6. Factors are then suggested to account for the general differences in performance of each of these vaccine classes, and suggestions are offered to assist in the development of effective protozoal vaccines for both animals and humans.

**Table 5.** Experimental animal and human immunizations using whole protozoal organisms^a^

Target	Animal	Pathogen	Immunization procedure	Adverse events	Challenge protocol	Results	Selected citations
Avian malaria	Canary	*Plasmodium relictum*	Sporozoites dissected from mosquitoes, held in physiological solution 12–48 h, and inoculated 1 × SC	In 24 birds, 1 had severe and 2 had weak parasitaemias when sporozoites held <24 h; 21 had no parasitaemia when held between 12–48 h	Birds exposed to infected mosquitoes at least 2 weeks after immunization	All 44 control birds developed malaria. Of 24 immunized birds: 1 had severe, 7 had mild and 16 had no parasitaemia	(Sergent and Sergent, 1910)
Falciparum malaria	Human	*Plasmodium falciparum*	Volunteers were bitten by 12–15 infected mosquitoes, 3 × , while on chloroquine	Malaria did not occur during this period	Bite of 5 mosquitoes, 2 m after 3rd exposure and 1 m after chloroquine	Malaria occurred in 5 of 5 control subjects but in 0 of 10 immunized subjects	(Roestenberg *et al.* 2009)
Avian malaria	Chicken	*Plasmodium gallinaceum*	Sporozoites dissected from mosquitoes and formalinized (a best protocol). 5 × 10^4^ IV, 3×	No illness reported.	Severe challenge with 500 sporozoites IV, 2 weeks after 3rd vaccination. Death as endpoint	All 20 control birds died but all 20 immunized birds survived with mild to minimal parasitaemia	(Richards, 1966)
Falciparum malaria	Human	*Plasmodium falciparum*	Sporozoites purified from mosquitoes, irradiated and cryopreserved. 1·35 × 10^5^ IV, 5× (the best protocol).	Injection site tenderness or bruising	Bite of 3 mosquitoes, ∼3 weeks after 5th vaccination	Malaria occurred in 5 of 6 control subjects but in 0 of 6 vaccinates	(Seder *et al.* 2013)
*Toxoplasma gondii* shedding by cats	Cat	*Toxoplasma gondii* strain T-263	Bradyzoites PO, 2 × , using sexually incompetent strain	No adverse event reported. No cat shed oocysts in feces following ingestion of T-263	Ingestion of heterologous bradyzoites in 200 cysts, 47 days after 2nd vaccination	All 6 unvaccinated control cats shed oocysts, but 0 of 24 vaccinated cats shed oocysts	(Freyre *et al.* 1993)
Toxoplasmosis transmission on farms	Cat	*Toxoplasma gondii* strain T-263	Cat trapping attempted during 7 visits to 8 farms over 3 years. Trapped cats administered live cryopreserved bradyzoites PO up to 2×	Not monitored.	n.a.	Significantly more cats were found shedding *T. gondii* oocysts prior to vaccinations. Progressive, statistically significant reductions of infection prevalence in pigs and in trapped mice on farms	(Mateus-Pinilla *et al.* 1999)
Cutaneous leishmaniasis	Human	Three *Leishmania* spp. isolates from Ecuador	ID, 2 × , killed promastigotes, mix of 3 isolates produced *in vitro*. BCG adjuvant	Minor local effects of inoculation and mild fever following 2nd vaccination were reported	n.a.	Within 1 year of vaccination, 2% of vaccinates *vs* 8% of controls had an episode of cutaneous leishmaniasis. Protective efficacy = 73%	(Armijos *et al.* 1998)
Cryptosporidiosis diarrhoea	Cattle	*Cryptosporidium parvum*	1 × , PO oocysts, killed by lyophilization, at 0–1 day age	None reported	10^5^ live oocysts PO at 7 days age	In 10 controls and 9 vaccinates, mean duration of diarrhoea was 4 *vs* 1·7 days, and mean duration of oocyst shedding 5·3 and 2 days, respectively	(Harp and Goff, 1995)
Histomoniasis	Turkey	*Histomonas meleagridis*	1 × , PO, live, *in vitro* high-passage attenuated trophozoites into the crop of 1 day old poults	No clinical signs nor reduced growth in vaccinated unchallenged birds compared with unvaccinated unchallenged controls	Intra-cloacal administration of 10^4^ low passage virulent trophozoites either at 15 or 29 days age	All 28 controls developed fatal histomoniasis. Only 4 of 14 vaccinated birds challenged at 15 days survived, but all 14 vaccinated birds challenged at 29 days remained clinically healthy and grew as well as uninfected controls	(Liebhart *et al.* 2010)

a All studies in this table used natural hosts and incorporated challenge controls or epidemiological comparisons.

**Table 6.** Representative comparisons of molecular malaria vaccine designs and their performance in artificially adapted animal models and human trials^a^

Vaccine design	Correlation between animal model and human results	Pathogen and host	Molecular details	Production	Inoculation	Results	Selected citations
Sporozoite recombinant antigen	Weak	*P. berghei* in mouse	CSP repetitive moiety (CS_170_) conjugated to BSA.	*In vitro* synthesis of CSP element	5× SC	Mice challenged with sporozoites 2 weeks after vaccination. Best protocol resulted in sterile immunity in 7 of 7 mice	(Reed *et al.* 1996)
		*P. falciparum* in humans	RTS,S fusion protein has half of CSP & entire surface ag of hepB virus	Recombinant ag from transfected yeast	3 × IM	This recombinant protein was first described in 1988. The best performing vaccine of this type. Latest field trials achieved 1 year efficacy of 44% in children and 30% in infants. Efficacy reduces over time despite frequent natural exposure	(Rutgers *et al.* 1988; Bejon *et al.* 2008; Agnandji *et al.* 2012; Olotu *et al.* 2013)
Erythrocyte stage recombinant antigen	Poor	*P. falciparum* in *Aotus* monkeys	Pf MSP-1	Recombinant ag from transfected *E. coli*	2 × IM	Protected 6 of 6 monkeys against uncontrolled parasitaemia following lethal (for naive monkeys) IV challenge with infected erythrocytes, 7 weeks after vaccination	(Darko *et al.* 2005)
		*P. falciparum* in humans	Pf MSP-1	As above	3 × IM	Vaccine efficacy < 15% in field trial. Children followed 6 m after last vaccination	(Ogutu *et al.* 2009)
Liver stage recombinant antigen	Poor	*P. falciparum* in *Aotus* monkeys	Pf LSA-3 long synthetic peptides	Recombinant ag from transfected *E. coli*	3 × SC	Protected 3 of 3 monkeys against parasitaemia from IV inoculation of *P. falciparum* sporozoites, 2 m after 3rd vaccination	(Perlaza *et al.* 2008)
		*P. falciparum* in humans	Pf LSA-NRC	As above	2 × IM	All 22 volunteers became parasitaemic after being bitten by 5 infected mosquitoes, 2 weeks after 2nd vaccination	(Cummings *et al.* 2010)
DNA using plasmids & live virus	Poor	*P. knowlesi* in rhesus monkeys	4 Pk ag & 3 cytokines encoded in plasmids, followed by live virus bearing the same Pk ag	Plasmid vectors & genetically modified live Vaccinia virus	Plasmid mix 4 × IM, then virus mix IM	Infectious challenge 2 weeks after viral boost. All 4 unvaccinated monkeys were treated for overwhelming parasitaemia. Of 11 vaccinated monkeys, sterile immunity occurred in 2 and spontaneous resolution of parasitaemia occurred in 7	(Rogers *et al.* 2002)
		*P. falciparum* in humans	Pf ME-TRAP in plasmid, followed by live virus with same ag. Or identical regimen substituting CS moieties for ME-TRAP	As above	Plasmid 2 × IM, then virus ID	7 of 8 volunteers develop parasitaemia after being bitten by 5 infected mosquitoes, 2 weeks following last vaccination with ME-TRAP. Parasitaemia was delayed 1 day. CS vaccine regimen had no protective effect	(Dunachie *et al.* 2006)

a To date, no protozoal vaccine based on these or similar molecular designs has achieved widespread success in humans or animals.

Throughout this manuscript, a vaccine is considered to have effective immunity or to demonstrate efficacy if it prevents infection or reduces disease incidence or severity, provided that such benefits extend for a useful period of time under field conditions. No assumptions are made about vaccine efficacy based on humoral, cellular, or innate immune response data, or experiments *in vitro* or within non-target animal models.

## PROS AND CONS OF VETERINARY VERSUS HUMAN VACCINE RESEARCH

### Comparative advantages for veterinary vaccine development

Vaccine development for animal diseases has the following advantages over vaccine development for human diseases.

#### Infectious challenge experiments

Development of vaccines for animals usually includes infectious challenge experiments using the natural animal host (Cornelissen and Schetters, 1996; Shkap and Pipano, 2000), enabling experiments with high predictive value to advance rapidly. Performing similar trials in humans has greater ethical and regulatory burdens and can be impossible or require compromise of experimental design. Therefore, initial efficacy data for humans may have to be acquired in the field under circumstances which can be difficult to control.

#### In vivo production of organisms

A second advantage in the development of animal protozoal vaccines is the option to manufacture vaccine organisms *in vivo*. Poultry coccidiosis vaccines are produced by infection of birds and collection and purification of organisms from their feces or intestines (Williams, 2002; Sharman *et al.* 2010). Many babesiosis vaccines are produced by infection of splenectomized animals and harvesting the infected erythrocytes (Callow *et al.* 1997). Although this is a clear manufacturing advantage, any vaccine that contains animal products is subject to marketing and regulatory disadvantages regarding international registration and importation.

#### Less rigorous regulation

Licensing requirements for human vaccines are stringent in modern industrial nations, including a requirement to demonstrate efficacy (see Gruber, 2011 for a review of US regulations). In comparison, animal vaccines have often appeared to have lower regulatory hurdles, as evidenced by commercial sales of various products prior to clear demonstration of efficacy (Choromanski and Block, 2000; Witonsky *et al.* 2004). Regulation of veterinary products in the European Union and the USA is becoming more rigorous (as reviewed by Schetters and Gravendyck, 2006).

### Comparative advantages for human vaccine development

#### Research funding

Naturally, public demand is much greater for treatments and vaccines to combat human diseases than for animal diseases. As a result, vastly more funding is available for human disease research of all types in comparison with animal disease research. For example, the 2012 budget for the US National Institutes of Health (NIH, 2012), which exists to perform and support human medical research, was $25·38 billion. In comparison, the US Department of Agriculture funds intramural animal health research via the Agricultural Research Service (ARS), and extramural animal health research via the National Institute for Food and Agriculture (NIFA). The ARS budget in 2012 that was allocated for ‘animal protection’, denoting animal disease research, was $0·08 billion (USDA, 2012). The last thorough analysis of NIFA's extramural research support was for a period ending in 2007 (NIFA, 2009), a year when approximately $0·05 billion was awarded for animal disease research. Although it isn't possible to compare with precision, NIH (human) and USDA (animal) health and disease research budgets clearly differ by orders of magnitude, and similar funding trends are apparent in the size and number of private charities and commercial industries that are devoted to human or animal health. This general funding environment affects all types of disease research, including efforts to develop protozoal vaccines.

#### Product pricing

Manufacturers of human vaccines benefit from significantly higher prices and increased demand for their products as a result of human priorities, medical insurance, government and school policies, and international humanitarian efforts. Pricing of livestock vaccines can be meagre in comparison; poultry coccidiosis vaccines are examples of products from which manufacturers need high volumes to compensate for exceptionally low prices (Williams, 2002).

## TYPES OF PROTOZOAL VACCINES IN USE

Most existing protozoal vaccines are for pathogens in the phylum Apicomplexa. Members of this phylum are obligate intracellular parasites that actively invade host cells and have both asexual and sexual reproductive cycles.

A small number of vaccines have been produced for non-Apicomplexan protozoa. These organisms only have asexual reproduction. Amastigotes of *Leishmania* spp. multiply within phagocytes of the vertebrate host although they do not invade non-phagocytic cells, and the promastigote stages replicate extracellularly within arthropod vectors. *Giardia lamblia* and *Tritrichomonas foetus* are strictly extracellular parasites.

Tables 1–4 provide examples of the types of protozoal vaccines that are in current use or have been in recent distribution.

### Human immunizations

#### Leishmanization

Old World and New World forms of cutaneous leishmaniasis are similar conditions caused by different species of *Leishmania*, which are transmitted by the bite of various phlebotomine sandflies. Infected bites may develop into chronic, ulcerated wounds (Ameen, 2010). Although most lesions ultimately resolve, disfiguring scars may result which can be particularly distressing when they occur on the face. Leishmanization is a controlled-exposure type of immunizing procedure that has been used to protect against cutaneous leishmaniasis (Table 1). Similar procedures were developed and used by several governments in the Middle East and central Asia, and related natural exposure practices (to sandflies) had been in prior use. In Iran, live virulent trophozoites of *Leishmania major* were produced by intraperitoneal mouse passage followed by limited axenic passage in broth (Nadim *et al.* 1983). The fresh live inoculum was administered intradermally or by scarification at a site in an arm or shoulder. If the procedure was a ‘take’, then a cutaneous lesion would develop at the inoculation site. This wound could endure months and in some cases longer than a year before healing with a scar. Immunized individuals were protected from disfiguring scars on the face that may result from natural exposure to *L. major*-infected sandfly vectors.

Quality assurance of these biological products was difficult. Adverse events were reported, most notably including persistent sores. Despite these problems, leishmanization was credited with greater than 80% reduction of disease prevalence (Nadim *et al.* 1997). A review by Dunning (2009) reported that leishmanization was recently employed in Uzbekistan.

#### Malaria fever therapy

To the author's knowledge, no other human protozoal immunizations have achieved widespread use, other than the possible historical inclusion of virulent malaria inoculations for fever therapy of neurosyphilis (‘general paralysis of the insane’) practiced in the early and mid-20th century. The intention was not to immunize against malaria, but rather to induce a high fever that would kill or control *Treponema pallidum* and thereby halt or reduce neurological impairment (as reviewed by Kragh, 2010) . Case reports have been compiled and examined for the effect upon the course of malaria itself when induced in patients more than one time, but highly varied treatment regimens and the understandable lack of controls make conclusions difficult (Collins and Jeffery, 1999).

### Immunization using controlled-exposure and infection-and-treatment protocols (see Table 1)

A logical improvement upon immunization with virulent organisms without treatment, where the infection is allowed to run its full course (controlled-exposure), is to infect patients with virulent organisms and then administer a prophylactic antimicrobial (infection-and-treatment). This practice is still widely used for East coast fever of cattle, and until recently was a common technique for control of poultry coccidiosis. An infection-and-treatment protocol was also recently used for experimental immunization against human malaria (Roestenberg *et al*., 2009; Table 5).

#### East coast fever of cattle

This is an economically important disease in eastern, central and southern regions of Africa. The causative organism is *Theileria parva*. Regionally varied species, subspecies, or variants of *T. parva* exist for which cross-protection may be poor (reviewed by Uilenberg, 1999). The parasite is transmitted by *Rhipicephalus* ticks and causes high mortality in *Bos taurus* cattle, less mortality in *Bos indicus* cattle, while native species of buffalo appear to be well-adapted natural reservoirs. Organisms transmitted by ticks invade, replicate within, and transform bovine lymphocytes, prior to invading erythrocytes from whence they are ingested by ticks; sexual recombination occurs in the tick prior to forming sporozoites within salivary glands.

These organisms are difficult to cultivate *in vitro* and reliable attenuation has not been achieved. Virulent seed stock organisms are used to infect cattle in the vaccine production process, which are then used to infect ticks. Organisms are harvested from tick salivary glands and are semiquantified, may be cryopreserved, and are used to immunize cattle in the field. Cattle are protected from disease by simultaneous injection of a long-acting formulation of oxytetracycline (Radley *et al.* 1975). Efficacy of this infection-and-treatment protocol is high and protection endures at least 3 years, but sterile immunity is not achieved. Immunized animals develop a carrier state with potential to transmit virulent organisms to ticks.

A multivalent vaccine (the Mugaga cocktail) (Patel *et al.* 2011), originally developed by the East African Veterinary Research Organization (now the Kenya Agriculture Research Institute), is licensed and being produced by a commercial enterprise for distribution in Kenya, Tanzania and Malawi (GALVmed, 2010). Different vaccine seed stocks are used in other regions. It is possible to immunize cattle in Zimbabwe using low-titration doses of the Boleni stock of *T. parva* without concurrent administration of antibiotics, attributed to its lower virulence (Latif and Hove, 2011).

The life cycle of the parasites of bovine theilerioses shares certain generalized features with that of malaria parasites, including sexual reproduction within a haematophagous arthropod vector, initial invasion within nucleated cells of the vertebrate host, and subsequent invasion of erythrocytes (Marquardt *et al.* 2000). Furthermore, infection-and-treatment protocols using the corresponding sporozoites induce protection against each of these diseases (Radley *et al.* 1975; Roestenberg *et al.* 2009).

#### Poultry coccidiosis

*Eimeria* spp. are coccidia of major economic importance to poultry industries around the world. These fecal-oral pathogens have a direct life cycle. Oocysts are excreted in feces and sporulate upon exposure to air. Ingested oocysts excyst in the gastrointestinal tract to release sporozoites, which invade host enterocytes. Each species undergoes a set number of asexual replication cycles within the intestinal tract, ultimately producing gametocytes that combine sexually and become oocysts (McDougald, 1998).

Live oocysts of various *Eimeria* spp. are purified from the feces of birds used for vaccine production. On farms, oral inoculation of hatchlings with mixed species of coccidial oocysts is used to induce protective immunity. Today, most if not all live coccidiosis vaccines are recommended to be applied without use of anticoccidial drugs, and thus they rely solely upon the birds’ ability to mount an effective immune response before serious superinfections can result from re-exposure to organisms in litter. Nevertheless, organism strains used in live vaccines are selected for their continuing sensitivity to anticoccidial drugs. Although now falling out of favour, a common practice was to vaccinate birds and then add low levels of coccidiostatic drugs to feed or water (i.e. infection-and-treatment) (reviewed by Williams, 2002).

Vaccinal protection from disease results from a combination of vaccine-induced immunity, immunological boosting from environmental re-exposure to vaccinal and wild type organisms, and displacement or dilution of coccidiostat-resistant strains in litter that otherwise may be prevalent (presumably this is in case anticoccidial treatment should become necessary). Because meat-producing birds have such short lifespans, induction of short-term immunity may be all that is required of a vaccine for broilers.

### Live attenuated vaccines (see Table 2)

A superior concept to controlled-exposure or infection-and-treatment strategies, when possible, is to develop vaccines that contain live but attenuated organisms that are unlikely to cause disease. The best performing protozoal vaccines have this design. Vaccinal organisms are sometimes manufactured *in vivo*, including many babesiosis vaccines and all attenuated coccidiosis vaccines; clearly, those production strategies are unsuitable for adaptation to purely human conditions. However, several attenuated vaccines are produced *in vitro*, have been attenuated using repeatable methods, and show little evidence of vaccine-associated adverse events; similar designs merit strong consideration for development of protozoal vaccines for human diseases.

#### Bovine tropical theileriosis

The causative organism, *Theileria annulata*, is a blood-borne apicomplexan parasite that is transmitted by an arthropod vector (*Hyalomma* ticks). Disease commonly occurs in highly susceptible *B. taurus* cattle in countries bordering the Mediterranean Sea and extending southward into Sudan and eastward into China and India.

Organisms are transmitted to cattle by ticks to invade, replicate within, and cause clonal expansion of macrophages and lymphocytes, prior to invading erythrocytes from whence they are ingested by other ticks. This species of *Theileria* is adaptable to *in vitro* culture within bovine macrophages or lymphoid cells. Many strains have become attenuated simply by prolonged passage in cell culture (Boulter and Hall, 1999; Gubbels *et al.* 2000). Attenuation has been associated with loss of expression of parasite-induced matrix metalloproteinases, which hinders systemic dissemination of organisms (Hall *et al.* 1999). Although vaccine strains may cause cryptic infections, latent organisms are incapable of transmission to ticks (Gubbels *et al.* 2000).

High rates of vaccine efficacy (>90%) and safety (100%) have been claimed (Zhang, 1997; Boulter and Hall, 1999) with little if any evidence of reversion to virulence. Immunity is cross-protective among strains (Boulter and Hall, 1999). Vaccinal protection endures at least 19 months in the field (Yin *et al.* 2008), although there is room to question whether the duration of vaccine efficacy might depend upon repeated exposure to infected ticks.

Similar vaccines for *Theileria lestoquardi* (*Theileria hirci*) are used in Iran, Iraq and Bulgaria to protect sheep from malignant ovine theileriosis (Hooshmand-Rad, 1985; Lawrence, 1997; Ali *et al.* 2008).

#### Toxoplasmosis abortion

An attenuated vaccine prevents *Toxoplasma gondii*-induced abortion in sheep (Buxton, 1993). This vaccine has been in continuous production since 1988 and is currently marketed in New Zealand and parts of Europe. It contains the S48 strain of *T. gondii*, which lost the ability to transform into the bradyzoite stage during years of twice-weekly intraperitoneal passage of tachyzoites in mice – such rapid passage did not allow time for stage conversion to form bradyzoites, and the strain lost this ability (Buxton and Innes, 1995). For the commercial production of the vaccine, the S48 strain is now maintained in tissue culture.

Ewes are vaccinated once, at least 3 weeks before breeding. Vaccination causes a transient fever and then the infection is cleared, unlike natural infections in which bradyzoites form latent intracellular cysts within brain and muscle. The inability to encyst makes the vaccine acceptable to use in a food-producing animal; otherwise, vaccine organisms in raw tissues could transmit infections to people and cats.

Vaccinal protection from toxoplasmosis abortion is strong against heterologous challenge with ingested oocysts (Wilkins *et al.* 1988), which has been tested out to 18 months post vaccination (Buxton *et al.* 1993). The manufacturer recommends revaccination in 2 years.

There are product warnings against vaccination of ewes during pregnancy, and because toxoplasmosis is a zoonotic disease there are further warnings about accidental human inoculation, particularly of pregnant women or immunosuppressed individuals. The author has been unable to find information to suggest that an adverse event may have occurred in people or that abortions in ewes may have been caused by inadvertent vaccination during pregnancy; this absence of published adverse events, after 25 years of commercial use, suggests (but does not prove) that despite the hypothetical concerns, any risks associated with the vaccine may in fact be minimal.

#### Bovine babesiosis

*Babesia* spp. are apicomplexan parasites of erythrocytes that are transmitted by tick vectors. Infection causes a febrile haemolytic disease. Morbidity and mortality can be particularly severe in *B. taurus* cattle.

Many bovine babesiosis vaccines are currently produced by governmental organizations around the world. Most are produced *in vivo* within splenectomized calves and contain *Babesia bovis, Babesia bigemina*, or both species. Many babesiosis vaccines may induce clinical disease; for example, a Cuban vaccine (Alonso *et al.* 1994) was reported to have a 2% rate of post-vaccination incidents. Vaccine risks are greater in adults (mirroring natural disease risks), so vaccinations are typically restricted to juveniles. Despite these limitations, babesiosis vaccines have been widely used in tropical and subtropical regions and have greatly reduced the incidence of disease losses (Callow *et al.* 1997).

A babesiosis vaccine currently in use in Argentina merits special attention because it is produced *in vitro* within bovine erythrocytes (Mangold *et al.* 1996), although it is not the only babesiosis vaccine to be cultured in this way (reviewed by Shkap and Pipano, 2000). Millions of doses of the combined *B. bovis* – *B. bigemina* vaccine have been used in Argentina, with failure of protection documented in 0·09% of vaccinated cattle (Echaide, 2008). The *B. bovis* fraction is known to have lost the ability to be transmitted to tick vectors.

In Europe, bovine babesiosis is caused by *Babesia divergens*, and vaccines have been produced for that pathogen (reviewed by Bock *et al.* 2008; Zintl *et al.* 2003). Many methods of attenuation and production were reported including *in vivo* within splenectomized calves and also within Mongolian gerbils (*Meriones unguiculatas*), or *in vitro* within erythrocytes. The use of gerbils for vaccine production was thought to have biosecurity and safety advantages over use of calves, however no more than 200 vaccine doses could be generated from a single gerbil. The apparent paucity of *B. divergens* vaccines available at present may have been influenced in part by the bovine spongiform encephalopathy epidemic in Europe and heightened concerns about disease transmission risks of bovine biological products.

An attenuated vaccine for *Babesia ovis* has been used to protect sheep in Bulgaria (Lawrence, 1997).

#### Precocious coccidiosis vaccines

An alternative to infection-and-treatment coccidiosis immunization protocols is the use of attenuated vaccines. The elegant ‘precocious’ method of attenuation was first described for *Eimeria tenella* (Jeffers, 1975), and the same method has been effectively applied to multiple species of poultry coccidia. Precocious strains of *Eimeria* spp. are selected by collecting the earliest forming oocysts from each infection, using those oocysts to infect the next subject, collecting the earliest oocysts again, and so on for multiple generations. Prepatent periods become shortened; this effect can be the result of the loss of one or more asexual cycles in the intestinal tract prior to gametogony (McDougald and Jeffers, 1976), or by a shortening of the time that each schizont takes to develop, and/or by a reduction in the size of schizonts and number of progeny within them (McDonald and Shirley, 2009).

Reduced asexual reproduction of precocious strains in the intestinal tract leads to less mucosal damage, fewer oocysts produced, and a corresponding reduction in the number of oocysts that accumulate in the environment (McDonald and Shirley, 2009). A manufacturing drawback is that because fewer precocious oocysts are produced in comparison with wild-type infections, a greater number of birds must be used for *in vivo* production of vaccinal oocysts and this increases production costs.

Precocious attenuation is a repeatable achievement and many stable strains have been developed. The prototype Wis-F strain of *E. tenella* could not be induced to revert to a longer prepatent period or virulence even when it was subjected to a relaxed selection protocol for 25 generations (Jeffers, 1975).

The precocious selection concept has recently been adapted to create *Eimeria* spp. vaccine strains for rabbits (Pakandl, 2005; Akpo *et al.* 2012), although it is unclear whether any have progressed to commercial use or regional distribution.

### Vaccines containing whole killed protozoa (see Table 3)

Only four examples were found of commercially marketed protozoal vaccines containing whole killed organisms. All of these vaccines incorporate adjuvants and require two initial immunizations. Although these products are safe, they induce at best only partial protection for a brief period of time. However, even brief protection may have useful niche applications, such as vaccination prior to the breeding season to reduce a sexually transmitted disease of cattle (trichomoniasis). Two of these products have been withdrawn from the market.

### Vaccines containing subunit antigens (see Table 4)

Only a few examples were found of commercially marketed protozoal vaccines based on defined or extracted antigens. All of the products in this class can be referred to as subunit vaccines; however, most of them are not recombinant antigen vaccines, because the vaccinal antigens are not derived from artificial synthesis or from transfection of other organisms such as bacteria, yeast, insect cells or viral vectors. All subunit protozoal vaccines incorporate adjuvants and require two or three initial immunizations.

Evidence of field efficacy has been published for two subunit protozoal vaccines (Leishmune and Coxabic). Unfortunately, the single example of a true recombinant antigen vaccine (Leish-Tec) has no documentation of field efficacy (from PubMed and Google searches or from the manufacturer's website).

#### Canine visceral leishmaniasis

Dogs and other canids are important reservoir hosts of *Leishmania infantum*, one of the pathogens of visceral leishmaniasis (canine and human). The pathogen is transmitted by sandflies when obtaining blood meals. In Brazil, a commercial vaccine for dogs (Leishmune) contains Fucose-Mannose-Ligand glycoprotein antigen derived from axenic culture of *L. donovani* (not *L. infantum*), administered with saponin adjuvant. This antigen complex plays a role in the entry of parasites into host phagocytes, a prerequisite for parasite replication and trafficking.

In field trials, vaccination with Leishmune reduced the incidence of disease in dogs over several years (Borja-Cabrera *et al.* 2002; Nogueira *et al.* 2005). Importantly, experimental evidence indicates that the vaccine helps to block transmission of *L. infantum* from dogs to sandfly vectors (Saraiva *et al.* 2006). These properties are consistent with epidemiological evidence associating vaccination of dogs with statistically significant reductions of human visceral leishmaniasis (Palatnik-de-Sousa *et al.* 2009).

In addition to vaccination of dogs, public health programmes in Brazil include identification and removal of *Leishmania*-infected dogs. This complicates epidemiological analysis, so further, public-sponsored investigations are merited to more clearly distinguish between the degree of human protection that is attributable to vaccination of dogs and to culling of infected dogs, respectively.

A similar *Leishmania* vaccine for dogs (CaniLeish) has been provisionally licensed in parts of Europe (EMA, 2011). The vaccine contains Excreted Secreted Proteins (ESP) of *L. infantum*. Pre-licensing experiments, using ESP in muramyl dipeptide as adjuvant, demonstrated protection from infectious challenge for at least 8 months (Lemesre *et al.* 2005), and a double-blind field trial of approximately 400 dogs showed 92% efficacy in preventing infections over a 2-year period (Lemesre *et al.* 2007). The provisionally licensed formulation of CaniLeish contains saponin adjuvant instead of muramyl dipeptide.

A third vaccine for canine leishmaniasis, licensed in Brazil since 2007 (Leish-Tec), is perhaps the only marketed recombinant antigen vaccine for any protozoal disease. It contains a *Leishmania* amastigote antigen (A2) purified from transfected *Escherichia coli*. A small trial showed partial short-term protection of vaccinated dogs from a severe infectious challenge (Fernandes *et al.* 2008). There appear to be no further published investigations of efficacy.

#### Coccidiosis of chickens

A subunit vaccine containing two native antigens extracted from *Eimeria maxima* macrogametocytes is marketed for vaccination of hens to provide passive immunization of chicks (Coxabic) (reviewed by Sharman *et al.* 2010). Organisms used in vaccine production are produced *in vivo* in chickens and are extracted from the intestinal tract. Maternal antibody is passed via the egg to hatchlings.

Passive immunity engendered by Coxabic is sufficient to enable offspring to perform well and to transition towards active immunity against mixed *Eimeria* spp. as a result of ubiquitous natural exposure. Vaccine efficacy has also been inferred from production data of large-scale field trials in which growth and mortality were similar between the offspring of vaccinated hens and the offspring of unvaccinated hens in which coccidiosis had been managed using traditional industry practices (i.e. prophylactic coccidiostatic drugs in feed, and/or use of live vaccines) (Wallach *et al.* 2008). Because of its manner of production and application, Coxabic is more expensive per dose than are other types of coccidiosis vaccines, however each vaccinated hen passes immunity to numerous offspring.

#### Canine babesiosis

A vaccine for canine babesiosis, containing soluble protein antigens derived from *in vitro* cultures of *Babesia canis* and *Babesia rossi*, was demonstrated to ameliorate the severity of infectious challenge with heterologous *B. canis* for up to 6 months after vaccination (Schetters *et al.* 2001, 2006). The infectious challenge in these experiments was severe, having been induced by intravenous inoculation of infected erythrocytes rather than by the bite of infected ticks; infectious challenge experiments that are overly harsh have the potential to mask the protective effect of a vaccine. In these experiments, haematocrit values dropped 54% in vaccinated dogs compared with a 65% drop in unvaccinated controls, and clinical scores also showed modest but statistically significant improvements (Schetters *et al.* 2006).

The vaccine was marketed in parts of Europe, but unfortunately there was no published epidemiological study and the product is no longer in production. Therefore, it is unclear whether the vaccine may have prevented or reduced transmission from ticks, or if the effects were limited to a reduction in the severity of disease as in the challenge experiments.

### Comparisons across vaccine classes

#### Propagation of organisms

Perhaps the single most obvious conclusion is that the production of all effective protozoal vaccines is dependent upon growth of protozoal organisms, either *in vitro* or *in vivo*; this includes production of organisms within arthropod vectors. Effective vaccines don't always contain whole organisms, but even native subunit vaccines require culture of organisms for extraction of antigens. The author has found only one commercially available recombinant antigen vaccine, but no evidence has been published to support the efficacy of that vaccine in the field. There are no licensed protozoal vaccines based on DNA or viral vectors.

This conclusion has a profound implication: for any protozoal disease to be controlled by a vaccine, methods must be developed to cultivate the pathogen. Although breaking this paradigm is a legitimate goal of protozoal vaccine science, vaccine designs with this goal carry a high risk of failure.

#### The value of living organisms

A single inoculation of living organisms, whether virulent or attenuated, tends to create strong, long-lasting protection against most protozoal diseases. The advantage of great efficacy is tempered by legitimate safety concerns, even though many attenuated vaccines have excellent safety records. A major rationale for research of novel protozoal vaccine designs for human diseases has been that live vaccines carry unacceptable risks; however, while many novel vaccines have been safe they have not been effective. Moving forward, new emphasis should be placed upon the development of novel methods to improve the safety of live vaccines, which can reasonably be expected to be effective.

#### Uses for short-term protection

Killed and subunit vaccines require adjuvants, booster inoculations, and annual or semiannual revaccination. The relatively short duration of protection is a disadvantage. However, residents of highly endemic regions may only need vaccinal immunity in the short term, and then naturally acquired immunity could take over as a result of frequent natural exposure. An example of this is vaccination of hens to provide passive protection of chicks from coccidiosis (Sharman *et al.* 2010).

#### Mucosal vaccines

In relation to mucosal pathogens, the endurance of effective immunity that may be attributed solely to live vaccination is unclear and is worthy of further research. For example, the true duration of effective immunity resulting solely from application of a poultry coccidiosis vaccine is obscured by the daily natural exposure of production birds to coccidia in litter (Williams *et al.* 2000). Although this distinction may be unimportant for the control of coccidiosis in poultry, the duration of protection of mucosal vaccines in the absence of frequent endemic exposure is an important consideration for development of future veterinary or human vaccines against diseases such as giardiasis, cryptosporidiosis, amoebiasis, microsporidiosis and trichomoniasis.

Parenteral vaccination with killed mucosal pathogens appears to provide modest, short-lived protection at most, and has been inadequately investigated (Lehmann and Lehmann, 2004; Baltzell *et al.* 2013).

## EXPERIMENTAL PROTOZOAL VACCINES

### Experimental vaccines containing organisms

Table 5 gives details of several noteworthy vaccination experiments that, although often highly effective, have not (or have not yet) resulted in a marketed vaccine. All of these experiments were based on live, attenuated, or whole-killed protozoal organisms. The results of these studies carry great predictive value because each was performed using a natural host–parasite relationship.

Two human malaria trials show protective efficacy for infection-and-treatment (Roestenberg *et al.* 2009) and whole-organism protocols (Seder *et al.* 2013); these experimental immunization protocols have counterparts among veterinary vaccines, and closely analogous experiments using avian malaria essentially predicted these results as much as a century earlier (Sergent and Sergent, 1910; Richards, 1966). Although an intravenously administered irradiated vaccine, recently examined by Seder *et al*. (2013), is described as being metabolically active, the experimental results compare closely with killed vaccines investigated in chickens (Richards, 1966). These investigations need to be progressed further, to include heterologous infectious challenges at meaningfully long intervals after immunization.

An attenuated vaccine to prevent cats from disseminating *T. gondii* oocysts was a remarkable scientific success but was not commercially viable. The vaccine was designed to reduce transmission from cats to other animals and humans. The T-263 strain of *T. gondii* was developed by chemical mutagenesis and selection for the inability to undergo sexual recombination (Frenkel *et al.* 1991). The orally administered vaccine demonstrated high efficacy in infectious challenge experiments of cats (Frenkel *et al.* 1991; Freyre *et al.* 1993). Additionally, in an ambitious 3-year field trial, cats were trapped and vaccinated on swine farms. The number of cats observed to be shedding oocysts and the *T. gondii* seroprevalence of pigs and rodents on farms decreased significantly (Mateus-Pinilla *et al.* 1999), consistent with decreased oocyst contamination of farms. Although effective, this vaccine would have been expensive to manufacture and distribute, and difficult to market.

Field evidence was obtained for efficacy of a vaccine to prevent human cutaneous leishmaniasis in Ecuador, which contained three local isolates of *Leishmania* spp. promastigotes that were phenol-killed and administered with BCG adjuvant. Vaccinated and control subjects were monitored for a year, and cutaneous leishmaniasis occurred in 2% and 8%, respectively (Armijos *et al.* 1998). The vaccine required a booster and annual revaccination (Armijos *et al.* 2003). Injection site swelling and mild fever were frequently observed especially following the second application; this could have been caused by reaction to *Leishmania* spp. antigens or to the BCG adjuvant. The vaccine does not appear to have been brought forward for commercial or governmental use. Another killed vaccine for human cutaneous leishmaniasis was recently trialled in Brazil and positive results were reported (Mayrink *et al.* 2013).

An attenuated vaccine for histomoniasis of turkeys and chickens has been developed by researchers in Austria. The vaccinal strain became attenuated when organisms were passaged every 2–3 days in axenic broth culture for 295 times. The vaccine is effective when administered into the crop or into the cloaca, and provides strong protection in challenge experiments, at least against low passage virulent organisms of the parent strain (Liebhart *et al.* 2010, 2013). Histomoniasis is re-emerging as a cause of poultry losses, in part because the prophylactic use of effective antimicrobials has been restricted or banned. Practical issues to overcome in order to commercialize this type of product will probably include the economics of transportation and administration of cryopreserved vaccines, which must be inexpensive to be used in poultry.

### Experimental molecular malaria vaccines

Table 6 compares results of animal models and human trials for several prominent types of experimental malaria vaccines. Malaria has been the dominant category of protozoal vaccine research in recent history. All of the vaccines in Table 6 were based on novel molecular designs.

In each comparison in Table 6, the positive results obtained in animal models exceeded the results obtained in humans. The following factors help explain the poor predictive value of those experiments: (1) Reliance upon artificially adapted host–parasite models of malaria; (2) Investigation of unreasonably short intervals between vaccination and infectious challenge experiments; (3) Use of homologous organisms in infectious challenge experiments; (4) Vaccine designs that disregard the history of veterinary protozoal vaccines. These four factors are discussed in greater detail in the following sections.

### The importance of animal model selection

All of the animal malaria models in Table 6 are artificial combinations of host and parasite. Natural malarias have a period of acute illness, during which mortality may occur in a minority of individuals, typically followed by recovery with prolonged splenomegaly, frequent development of latent infections, and the possibility of disease relapse (Garnham, 1966; Miller *et al.* 1994). Metaphorically, there is a standoff between host and parasite rather than absolute victory for either one. In contrast, artificially adapted malaria models tend to behave in a ‘winner-takes-all’ manner that does not resemble naturally evolved malaria relationships.

Laboratory rodent-adapted *Plasmodium* spp. are natural parasites of *Thamnomys* and *Grammomys* spp. of thicket rats (Vincke and Lips, 1948; Cox, 1988), native to montane forests of central Africa. The ancestral lineage of these rodents diverged from that of the house mouse over 10 million years ago (Lecompte *et al.* 2008). The natural hosts are well adapted and do not suffer high mortality (Garnham, 1980). In contrast, experimental malaria in laboratory mice has high mortality, with many mouse–parasite combinations being completely lethal. Rescue of infected mice tends to cause sterile recovery (i.e. without chronic or latent infections) (Ishih *et al.* 2013). This is a winner-takes-all scenario.

Natural hosts of *Plasmodium knowlesi* include long-tailed macaques (*Macaca fascicularis*) and pig-tailed macaques (*Macaca nemestrina*), in which the results of infection are variable and have low mortality (Butcher *et al.* 2010). Rhesus monkeys (*Macaca mulatta*) are not natural hosts of *P. knowlesi*, and infection in them induces fulminating parasitaemia usually resulting in death (Collins, 1988), similar to *Plasmodium berghei* infection of mice. This is another winner-takes-all scenario.

Infection of owl monkeys (*Aotus* spp.) with the human pathogen *Plasmodium falciparum* creates another winner-takes-all scenario (Sadun *et al.* 1969).

In artificial winner-takes-all relationships, relatively minor treatments may cause a radical shift between the only two major outcomes, death or sterile recovery. Researchers may mistakenly believe that lethal models of malaria are severe tests for any vaccine, and that survival after vaccination is therefore evidence of great efficacy. In practice, a vaccine that reduces infection or moderates disease in any naturally evolved host–parasite relationship is more predictive of what may be expected in other animals with similar parasites, presumably including humans. A vaccine that ameliorates the course of naturally evolved malarias in *Thamnomys* rodents, pig-tailed macaques, canaries, or chickens, should be expected to have greater predictive value for human malaria than do vaccines that block mortality in mice, owl monkeys, or in other artificially adapted animal models.

### Short testing intervals

Data for the protective efficacy of most vaccines should be assessed over a range of intervals that extend into a useful duration. Short-lived immunity may be adequate for poultry coccidiosis or seasonal breeding of cattle; however, vaccines for systemic illnesses such as malaria should be tested at least to 6 months, and preferably to a year or more.

### Homologous vs heterologous infectious challenge

To be effective in the field, vaccines need to protect against a variety of organism isolates. When possible, vaccines should be tested by challenge with heterologous organisms, rather than with the same strain from which the vaccine was manufactured. Research efforts should include acquisition of varied isolates for any pathogen under investigation. Ultimately, efficacy must be assessed in field trials within endemic regions.

### Vaccine designs that are useful in animals

There is a reason why veterinary medicine hasn't created a bevy of recombinant antigen vaccines for the prevention of protozoal diseases of animals – they haven't been sufficiently effective.

This is not to imply that such vaccine designs could never work, or that transformational new knowledge will never open a floodgate of effective vaccines based on novel technological advancements. Potentially transformative but technologically difficult and unproven vaccine designs should be investigated first in naturally occurring diseases of animals, not in artificial models. Translation of a novel design principle to human vaccines should be attempted after it has been shown to be effective against a natural animal–parasite relationship. If science cannot create an effective recombinant antigen or plasmid vaccine to prevent infection of (for example) rats with *T. gondii*, deer mice with *Babesia microti*, or sparrows with *Plasmodium relictum*, then it seems rather naive to expect such designs to be effective against human protozoal diseases.

Funding for veterinary medical research is paltry in comparison to human disease research. Human medical research agencies should fund research of protozoal diseases of animals in order to develop improved vaccine methods and investigate new paradigms. At the same time, protozoal vaccines for people should be developed by adapting currently effective techniques from veterinary medicine.

## ONE MEDICINE

The concept of ‘One Medicine’ includes the beneficial flow of knowledge and techniques from human medicine to veterinary medicine, and from veterinary medicine to human medicine. However, because of the dominance of human medical research funding, the flow of information moves predominantly from human medicine to veterinary medicine. Human medicine is missing significant benefits that could be had by paying greater attention to veterinary knowledge and by supporting opportunities to investigate naturally occurring diseases of animals. This missed opportunity is vividly illustrated by the discordance between development of veterinary protozoal vaccines, of which there are many, and human protozoal vaccines, of which there are none.
